# Significance of the Boston Cognitive Assessment in patients with chronic post-stroke cognitive impairment

**DOI:** 10.3389/fneur.2025.1690494

**Published:** 2025-11-28

**Authors:** Xiao Yin, Haiying Zhu, Panpan Ji, Beixuan Wu, Yi Zhang

**Affiliations:** 1 Department of Rehabilitation, Changzhou First People’s Hospital and Third Affiliated Hospital of Soochow University, Changzhou, Jiangsu, China; 2 Department of Rehabilitation, Jiangsu Second Rongjun Hospital, Wuxi, Jiangsu, China; 3 Changzhou De’an Hospital, Changzhou, Jiangsu, China

**Keywords:** Boston Cognitive Assessment, brain health, stroke, impairment, cerebral vascular disorder

## Abstract

**Background:**

The Boston Cognitive Assessment (BoCA) is an online, self-administered, remote cognitive screening tool for the early detection and long-term monitoring of health changes in the brain of aging populations. This study aimed to evaluate the reliability and validity of the Mandarin version of the BoCA in Chinese patients with stroke, thereby providing a reference for important clinical applications.

**Methods:**

This study included 120 patients with chronic stroke and 120 healthy controls. All participants completed the Mini-Mental State Examination (MMSE), Montreal Cognitive Assessment (MoCA), and Mandarin versions of the BoCA. Assessments were spaced a minimum of 60 min apart. For the test–retest reliability analysis, 120 patients with stroke were retested on the BoCA after 1 week. The receiver operating characteristic (ROC) curves were generated to assess diagnostic performance.

**Results:**

Among the stroke group, the BoCA showed appreciable internal consistency (Cronbach’s *α* = 0.808) and significant test–retest reliability (intraclass correlation coefficient, ICC = 0.895, *p* < 0.001). The BoCA subscales demonstrated moderate-to-strong correlations with the total score (*r* = 0.546–0.770), supporting adequate content validity. The BoCA total scores were strongly correlated with the MMSE (*r* = 0.829, *p* < 0.001) and MoCA (*r* = 0.848, *p* < 0.001) scores, demonstrating adequate criterion-related validity. Exploratory factor analysis (EFA) of the BoCA tasks revealed one robust factor accounting for a plurality (i.e., 46.9%) of the total variance, indicating sufficient construct validity. An ROC analysis revealed comparable diagnostic performance for the BoCA (area under the curve, AUC = 0.823), MMSE (AUC = 0.836), and MoCA (AUC = 0.818). A BoCA score of 23.5 distinguished the stroke group from the control group with 81.7% sensitivity and 69.2% specificity.

**Conclusion:**

The Mandarin version of the BoCA exhibits significant reliability and validity and functions effectively as a supplementary measure for cognitive assessment in stroke survivors.

## 1 Introduction

Stroke, a prevalent cerebral vascular disorder, is a global health crisis and is the second leading cause of mortality and the primary driver of adult disability (1). China, the world’s most populated country, has the highest incidence rates and is disproportionately affected by rapid societal aging (2). Nearly one-third of patients with stroke experience post-stroke cognitive impairment (PSCI) as a chronic sequela (3). PSCI is defined as persistent cognitive deficits occurring post-stroke, lasting at least 6 months (4), and frequently progressing over time. This progression severely compromises functional independence, diminishes workforce participation, and hinders routine functional activities, thereby exacerbating caregiver dependence and increasing mortality risk. Therefore, longitudinal follow-up cognitive ability testing in patients with post-stroke sequelae has major clinical implications.

The Mini-Mental State Examination (MMSE) (5) and the Montreal Cognitive Assessment (MoCA) (6) are the most widely used cognitive screening tools. Both tools cover core cognitive domains (attention, executive function, memory, language, and visuospatial ability). Although validated for PSCI screening in Chinese stroke survivors (7–9), both the MMSE and MoCA have clinically significant limitations. In terms of intrinsic psychometric characteristics, both tools have significant deficiencies. The MMSE has limited sensitivity in detecting mild cognitive impairment and early vascular cognitive dysfunction, often failing to identify the typical features of PSCI, including executive function and visuospatial dysfunction (10). Although the MoCA aims to address this issue through higher sensitivity, both tools exhibit marked dependence on education level and cultural background, underestimating cognitive function in individuals with lower educational attainment (8). In addition to these psychometric limitations, practical applications also face major challenges. Both tools require in-person administration at designated healthcare facilities for supervised paper-and-pencil testing, thereby limiting accessibility for patients facing transportation barriers or pandemic restrictions (10). Their dependence on specialized medical personnel for supervised testing often induces assessment anxiety (the white-coat phenomenon), whereas lengthy administration consumes substantial resources (10, 11). Moreover, fixed content causes practice effects that compromise longitudinal tracking accuracy during repeated administrations (12).

The barriers to conventional PSCI screening, particularly mobility limitations and psychosocial reluctance, create marked gaps in detection. Developing validated, cost-effective, and remote cognitive assessments could address this unmet need for homebound stroke survivors. The Telephone Interview for Cognitive Status (TICS), the Telephone-Montreal Cognitive Assessment (T-MoCA), and MoCA 5-min protocol have been validated as cognitive screening tools for patients with stroke, demonstrating adequate reliability and validity (10, 13, 14). However, owing to the limitations of the form, telephonic assessments fundamentally exclude the assessment of visuospatial construction (cannot administer clock-drawing or figure-copying tasks), executive function (cannot evaluate trail-making or design fluency), and expressive speech (lacks visual confrontation naming evaluation). This inherent domain restriction produces a narrower cognitive profile than traditional paper–pencil assessments, rendering them inadequate for comprehensive large-scale population screening.

With advancements in computer and tablet technology, Gold et al. (15) developed the Boston Cognitive Assessment Scale (BoCA), a 10-min self-administered online test for cognitive assessment. BoCA incorporates the following characteristics: (1) tele-assessment capability—which enables remote cognitive testing without geographical constraints; (2) self-administration—which requires no clinician supervision; (3) practice-effect mitigation, which employs randomized non-repeating stimuli across sessions; and (4) cross-cultural adaptability, which offers multi-language versions for global deployment. Studies have demonstrated that BoCA shows adequate reliability and validity in older adult populations in the United States, strongly correlates with the MoCA and TICS, and has high sensitivity and specificity for detecting mild cognitive impairment (MCI) (15–17). The Italian version of the BoCA test exhibited good validity, feasibility, and good discriminant validity comparable to the MoCA (12). To date, the BoCA has not been implemented for cognitive impairment screening in China, and globally, no validation studies have been conducted specifically in stroke populations.

In this study, we examined the reliability and validity of the Mandarin version of the BoCA in Chinese patients with stroke and compared its diagnostic performance for PSCI with those of the MMSE and MoCA.

## 2 Materials and methods

### 2.1 Patients

The undergraduate research project has been approved by the Ethics Committee of Jiangsu Second Rongjun Hospital [Ethics Review No. (2024) 003]. A total of 120 home-based patients with stroke who were followed up at the Department of Rehabilitation Treatment, Jiangsu Second Rongjun Hospital, from July 2024 to June 2025 were enrolled in the experimental group (stroke group).

The inclusion criteria were as follows:

1. Stroke diagnosis was confirmed using cranial computed tomography (CT) or magnetic resonance imaging (MRI), complying with the 2023 Chinese Stroke Diagnosis Guidelines.
2. Age ≥18 years.
3. More than 6 months since the last stroke occurred.
4. Preserved consciousness and ability to comply with testing protocols.
5. Adequate audiovisual, motor, and language comprehension for cognitive assessment.
6. Ability to provide written informed consent. In context, consent was obtained from the participant or their legally authorized representative.

The exclusion criteria were as follows:

1. Severe cognitive impairment, profound visual/hearing impairment, or global aphasia precluding test completion.
2. Pre-stroke history of cognitive/psychiatric disorders (AD8 score ≥2).
3. Current use of cognition-impairing medications or psychoactive substances.
4. Severe systemic comorbidities.

A separate group of 120 demographically matched healthy community-dwelling residents served as controls, with matching criteria for age, sex, and years of education. The exclusion criteria were individuals with a history of brain injury (brain trauma or stroke), individuals with a history of cognitive decline (dementia, mental disorders, or hypothyroidism), individuals who use drugs that may affect cognitive function, and individuals who refuse to participate.

Using convenience sampling, all participants were enrolled, and the MMSE, MoCA, and Mandarin BoCA were administered. Assessments were spaced at least 60 min apart. For the test–retest reliability analysis, 120 patients with stroke completed a repeat BoCA assessment 1 week later. The MMSE and MoCA were administered by a professionally trained rehabilitation physician. The BoCA was self-administered by participants using electronic tablet devices through a web-based platform. The BoCA test was automatically scored, and the total and subtest scores were obtained immediately after administration.

### 2.2 Measures

The Mini-Mental State Examination (MMSE) (5, 18) includes six cognitive domains: orientation, memory, attention, calculation, language, and visuospatial skills. Higher scores indicate better cognitive performance, with a maximum score of 30.

The Montreal Cognitive Assessment (MoCA) (6, 19) includes seven cognitive domains: visuospatial and executive functions, naming, attention, language, abstraction, memory, and orientation. Higher scores indicate better cognitive performance, with a maximum score of 30.

The BoCA (15) is a 10-min, self-administered, remote online test. The BoCA includes eight subscales that evaluate various cognitive domains: memory/immediate recall, memory/delayed recall, executive functions/clock-drawing test, visuospatial reasoning/mental rotation, mental mathematics, attention, language/prefrontal synthesis, and orientation. Higher scores indicate better cognitive performance, with a maximum score of 30.

### 2.3 Evaluation process

During home visits or outpatient follow-up visits by professionally trained rehabilitation physicians, MMSE tests were completed first, followed by MoCA tests after an interval of at least 2 h. The entire process was conducted in a quiet, undisturbed environment. The rehabilitation physician explained the purpose, content, and process of the assessment to the participants and scored them.

Thereafter, the participants and their families were trained on the use of BoCA and provided with the secure test link (URL). The researcher’s account was used to demonstrate the operation of the assessment page. The BoCA can be used on smartphones and tablets. Participants unable to access the start page of the test were assisted by family members.

Within 48 h after returning home, participants independently completed the online BoCA in a quiet environment. At least 7 days after the initial evaluation, the stroke group completed the BoCA re-evaluation using the same method. After completing the BoCA assessment, the participants reported the test date, total score, and code to the researcher via phone or SMS, which the researcher then recorded in their account.

### 2.4 Statistical analyses

Statistical software (SPSS 27.0) was used to analyze the data. In this study, the Kolmogorov–Smirnov test was used to assess the normal distribution of the data. Normally distributed continuous variables are presented as mean ± standard deviation (SD). Non-normally distributed continuous variables are expressed as median (interquartile range) and were compared using the rank-sum test. Categorical variables are summarized as the number of cases (percentage). Cronbach’s *α* was used to determine internal consistency. Intraclass correlation coefficient (ICC) analysis was used to evaluate test–retest reliability. Spearman’s correlation coefficient was used to examine the content validity. Exploratory factor analysis was conducted to analyze construct validity. Comparisons between the MMSE and MoCA scales were made, and correlation coefficients were calculated to assess criterion-related validity. Diagnostic performance was assessed using receiver operating characteristic (ROC) curve analysis based on the area under the curve (AUC) values. Statistical significance was set at a *p*-value of <0.05.

## 3 Results

### 3.1 Descriptive analysis

Demographic characteristics for the stroke and control groups are presented in Table 1. No significant differences were found between the stroke and control groups in terms of sex, age, or educational level (*p* > 0.05). The results demonstrated statistically significant differences in the MMSE, MoCA, and BoCA scores between the stroke and control groups (*p* < 0.001), with the stroke group exhibiting significantly lower total scores across all three cognitive assessments than the control group.

**Table 1:** Descriptive statistics for patients with stroke and controls.

Variables	Stroke group	Control group	*p*-value
Age, mean years (SD)	74.6 (11.0)	73.2 (9.1)	0.110
Education	11 (9, 13)	12 (9, 13)	0.458
Sex, male, *N* (%)	74 (61.7)	65 (54.2)	0.239
Clinical features			
MMSE total score	25 (19,27)	26 (22, 29)	<0.001
MoCA total score	17 (11.25, 22)	26 (22, 26)	<0.001
BoCA total score	18 (13, 23)	25 (22.5, 27)	<0.001

SD, standard deviation; MMSE, Mini-Mental State Examination; MoCA, Montreal Cognitive Assessment; BoCA, Boston Cognitive Assessment.

### 3.2 Reliability analysis

The internal consistency of the eight BoCA subscales in stroke patients was assessed using Cronbach’s *α*. The results indicated a significant overall internal consistency (Cronbach’s *α* = 0.808). A sensitivity analysis was then conducted by systematically removing one subscale at a time and recalculating Cronbach’s *α* for the remaining seven standardized subscales (Table 2). The resulting Cronbach’s *α* for the seven subscale composites was positive, indicating high and stable internal consistency (Cronbach’s *α* > 0.78).

**Table 2:** Internal consistency of the eight BoCA subscales in patients with stroke.

BoCA subscales	Cronbach’s *α* if parameter deleted
Memory/immediate recall	0.788
Language	0.800
Mental rotation	0.795
Clock test	0.789
Attention	0.778
Mental mathematics	0.763
Orientation	0.789
Memory/delayed recall	0.781
BoCA total score	0.808

BoCA, Boston Cognitive Assessment.

The test–retest correlation coefficient for the BoCA total score was 0.895 (95% CI: 0.844–0.930), indicating excellent test–retest reliability of the BoCA in patients with stroke. As shown in Table 3, all individual BoCA subscales exhibited significant test–retest correlations.

**Table 3:** Test–retest reliability of BoCA in patients with stroke.

BoCA subscales	ICC	95% CI	*p*-value
Memory/immediate recall	0.453	0.264–0.599	<0.001
Language	0.368	0.202–0.553	<0.001
Mental rotation	0.497	0.254–0.601	<0.001
Clock test	0.466	0.286–0.616	<0.001
Attention	0.659	0.526–0.765	<0.001
Mental mathematics	0.693	0.569–0.789	<0.001
Orientation	0.655	0.489–0.744	<0.001
Memory/delayed recall	0.662	0.518–0.761	<0.001
BoCA total score	0.895	0.844–0.930	<0.001

BoCA, Boston Cognitive Assessment; ICC, intraclass coefficient; CI, confidence interval.

### 3.3 Validity analysis

The criterion-related validity of the BoCA was assessed by correlation with MMSE and MoCA scores. The BoCA total scores were highly correlated with MMSE scores (*r* = 0.829, *p* < 0.001) and MoCA scores (*r* = 0.848, *p* < 0.001), indicating that the BoCA in patients with stroke has appreciable criterion-related validity.

Spearman’s correlation coefficients for the BoCA subscales and total scores ranged from 0.546 to 0.770. All subscales demonstrated significant and moderately positive correlations with the total score. Table 4 shows that the BoCA has considerable content validity in patients with stroke.

**Table 4:** Correlation analysis of BoCA subscales and the total score.

BoCA subscales	Parameter-total correlation	*p*-value
Memory/immediate recall	0.657	<0.001
Language	0.546	<0.001
Mental rotation	0.582	<0.001
Clock test	0.649	<0.001
Attention	0.717	<0.001
Mental mathematics	0.770	<0.001
Orientation	0.619	<0.001
Memory/delayed recall	0.731	<0.001

BoCA, Boston Cognitive Assessment.

**Figure 1:**
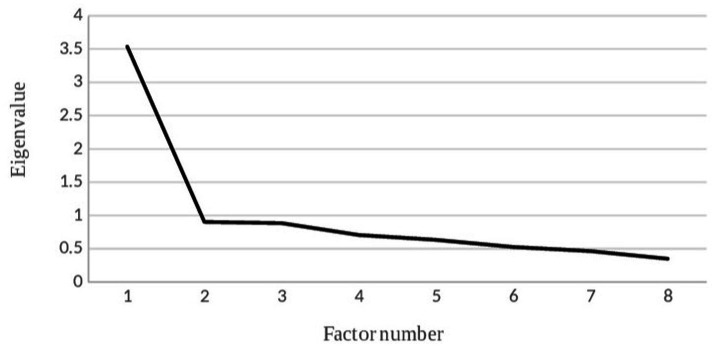
Scree plot.

The Kaiser–Meyer–Olkin (KMO) measure was 0.847, and Bartlett’s test of sphericity yielded *χ*^2^ = 212.888 (*p* < 0.001), indicating that the scale was suitable for factor analysis. Factor analysis of the eight BoCA subscales yielded factor 1 with an eigenvalue of 3.755, accounting for 46.9% of total variance. Factor 2 had an eigenvalue of 0.932, accounting for 11.7% of the total variance. The eigenvalues of the remaining six factors were less than 0.8. As shown in Table 5, factor 2 contributed minimally to the variance. The scree plot (Figure 1) showed a clear leveling off (elbow point) after a factor of 1. As shown in Table 6, all subscales had factor loadings >0.6 on factor 1. Therefore, only factor 1 was retained. We identified this single factor as “Global Cognitive Function,” encompassing all eight subscales.

**Table 5:** Factor analysis results.

Factor	Eigenvalue	Variance contribution rate (%)	Cumulative variance contribution rate (%)
1	3.755	46.9	46.9
2	0.932	11.7	58.6
3	0.735	9.2	67.8

**Table 6:** Factor loading matrix of BoCA.

BoCA subscales	Factor 1 (46.9%)
Memory/immediate recall	0.724
Language	0.603
Mental rotation	0.617
Clock test	0.718
Attention	0.731
Mental mathematics	0.785
Orientation	0.602
Memory/delayed recall	0.677

BoCA, Boston Cognitive Assessment.

### 3.4 Diagnostic accuracy

**Figure 2:**
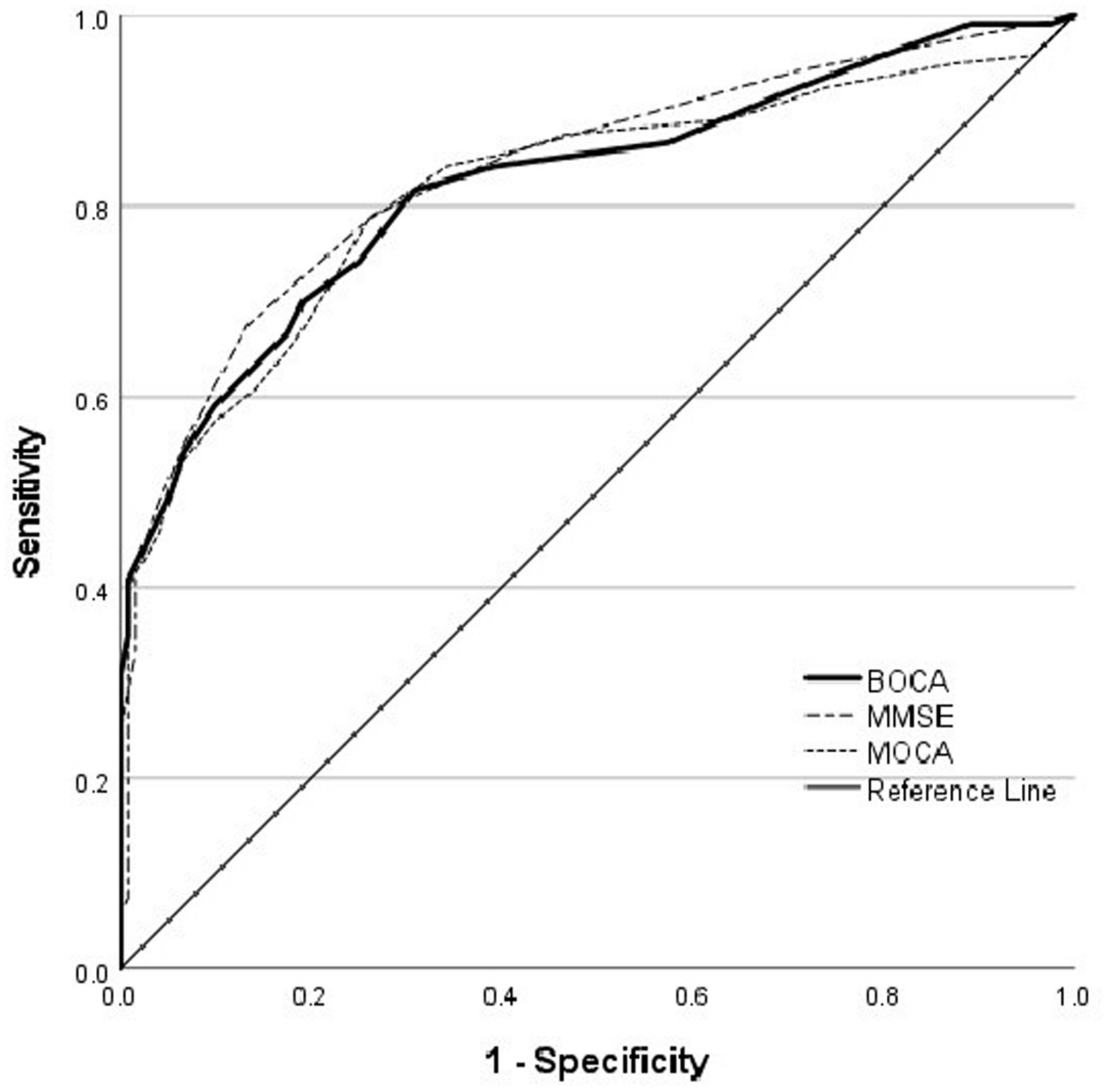
ROC for MMSE, MoCA, and BoCA scales. ROC, receiver operating characteristics; MMSE, Mini-Mental State Examination; MoCA, Montreal Cognitive Assessment; BoCA, Boston Cognitive Assessment.

The receiver operating characteristic (ROC) analysis revealed comparable diagnostic accuracies among these tests, as evidenced by the area under the curve (AUC) (MMSE, 0.836; MoCA, 0.818; BoCA, 0.823). An MMSE score of 26.5 distinguished the stroke group from the control group with 67.5% sensitivity and 86.7% specificity. A MoCA score of 22.5 distinguished the stroke group from the control group with 78.3% sensitivity and 74.2% specificity. A BoCA score of 23.5 distinguished the stroke group from the control group with 81.7% sensitivity and 69.2% specificity (see Figure 2 and Table 7).

**Table 7:** ROC analysis results for MMSE, MoCA, and BoCA: AUC, optimal cutoff points, and corresponding sensitivity and specificity.

Cognitive assessment tools	AUC	The largest Youden index	Cutoff points	Sensitivity	Specificity
MMSE	0.836	0.542	26.5	0.675	0.867
MoCA	0.818	0.525	22.5	0.783	0.742
BoCA	0.823	0.509	23.5	0.817	0.692

ROC, receiver operating characteristics; MMSE, Mini-Mental State Examination; MoCA, Montreal Cognitive Assessment; BoCA, Boston Cognitive Assessment; AUC, area under the curve.

## 4 Discussion

Cognitive trajectories in post-stroke impairments show dynamic progression. Quarterly cognitive surveillance after stroke is warranted (2), especially considering the significant risk of dementia within 5 years (20). Systematic screening thus represents a care imperative for optimizing rehabilitation, enhancing quality of life, and reducing socioeconomic burden. Leveraging its advantages of convenience, decentralized testing, resistance to confounding factors, and linguistic inclusivity, the BoCA shows considerable potential for addressing the core challenges of accessibility, efficiency, and reliability inherent in traditional PSCI screening scales. Therefore, this study examined the reliability and validity of the Mandarin version of the BoCA for cognitive screening in a Chinese population with stroke and assessed its diagnostic efficacy.

This is the first study to use the Mandarin version of the BoCA for cognitive screening in China and the first to validate its use specifically in a stroke population. This study validated the BoCA in 120 patients with stroke and 120 age-, sex-, and education-matched controls. Test scores were significantly different between patients with stroke and those in the control group (*p* < 0.001), suggesting a good discriminative ability.

Internal consistency of the BoCA subscales was high in patients with stroke (Cronbach *α* = 0.808). Cronbach’s *α* coefficient observed in this study falls between the values reported by Gold et al. (15) (0.81) and Vyshedskiy et al. (16) (0.79). Removing any single parameter from the BoCA could lower the overall Cronbach’s *α* coefficient, although the value remained consistently above 0.78. This decrease was comparatively smaller when parameters related to language function were removed. This result indicates that the Mandarin BoCA exhibits adequate and stable internal consistency for assessing cognition in Chinese patients with stroke. The total score on the Mandarin BoCA had a test–retest reliability of 0.895 [95% confidence interval (CI): 0.844–0.930]. The test–retest coefficients observed in this study were between the values reported by Gold et al. (15) (0.89) and Vyshedskiy et al. (16) (0.94), indicating excellent long-term stability of the BoCA. This stability may be attributed to several factors. First, both tasks adopt a single-choice mode with a small number of options (2, 3), resulting in a high probability of guessing and introducing significant random measurement errors. Second, the highly heterogeneous questions used to reduce learning effects may measure cognitive sub-components that are not completely identical, reducing the homogeneity and comparability of scores across questions. Finally, the BoCA tasks require a complex paradigm of ‘visual search after listening to instructions,’ the successful execution of which depends on a complete perceptual attention executive functional chain. In this study, basic perceptual disorders such as unilateral neglect were not screened; therefore, it cannot be ruled out that the visuospatial attention deficit in the stroke group may have contaminated the test–retest stability. To improve the test–retest reliability of some sub-items, future assessments could incorporate interference options, simplify question stems, and screen for unilateral neglect in advance.

For item–total correlation (ITC) analyses, all subscales demonstrated significant moderately positive correlations with the total score (ITC: 0.546–0.770, *p* < 0.001). The highest ITC for mental mathematics was 0.770, and the lowest ITC for language was 0.546. The results showed that the Mandarin BoCA has good content validity in patients with stroke. In further support of BoCA’s validity, the exploratory factor analysis (EFA) yielded a single factor explaining a plurality of the variance in the scores of patients with stroke. This factor had an eigenvalue of 3.755 and a variance contribution rate of 46.9% of the total variance. This finding is consistent with previous studies. Gold et al. (15) identified a factor with an eigenvalue of 3.33 and a variance contribution rate of 42%, while Vyshedskiy et al. (16) reported a factor with an eigenvalue of 4.14 and a variance contribution rate of 51.76%. This factor reflects global cognitive functioning, indicating that the test’s internal structure matches the construct intended to be measured. Additionally, correlational analyses revealed strong correlations between BoCA and MMSE scores (*r* = 0.829, *p* < 0.001) and between BoCA and MoCA scores (*r* = 0.848, *p* < 0.001). These significant positive correlations demonstrate that the BoCA scale has sufficient criterion-related validity.

The MMSE, MoCA, and BoCA demonstrated comparable AUC values (MMSE, 0.836; MoCA, 0.818; and BoCA, 0.823), indicating equivalent diagnostic performance for detecting cognitive impairment in patients with stroke. Analysis of the ROC curve revealed that the Youden index was maximized at a cutoff score of 26.5 for the MMSE, 22.5 for the MoCA, and 23.5 for the BoCA. Therefore, an MMSE score of ≤26 points indicated impairment, consistent with widely applied diagnostic criteria (21), while an MoCA score of ≤22 points indicated impairment, consistent with Wang’s (2) findings. A BoCA score of ≤23 points was indicative of cognitive impairment. At the optimal cutoff, the MMSE achieved a sensitivity of 0.675 and a specificity of 0.867, while the MoCA achieved a sensitivity of 0.783 and a specificity of 0.742. The BoCA achieved a sensitivity of 0.817 and a specificity of 0.692, indicating greater sensitivity compared with the other tools, which makes it particularly suitable for large-scale screening aimed at targeting the early detection of PSCI in patients with stroke. However, BoCA’s lower specificity results in a higher false-positive rate.

As a preliminary assessment of the Mandarin version of the BoCA, this study had certain limitations. Heterogeneity in the types of strokes was unaccounted for, as we included patients with both ischemic and hemorrhagic strokes. These conditions exhibit marked differences in pathological mechanisms and cognitive impairment profiles. The current findings did not differentiate how subtypes of strokes influenced scale efficacy. Future studies should conduct subgroup analyses using validated classification systems (e.g., TOAST/OCSP typology). Second, stratification according to disease severity was not performed. The absence of grading based on standardized measures (e.g., NIHSS for neurological deficit or mRS for disability levels) may introduce bias toward mild-to-moderate cases. Urgent validation across the severity strata is required to determine the sensitivity gradients of the cognitive scales. In addition, there is an inadequate adjustment for educational adaptation. Standardized English scales use linguistic adaptations that are theorized to minimize educational bias. Without a stratified analysis across education tiers, we could not validate these properties in our population.

## 5 Conclusion

This study validated the effectiveness of the BoCA for screening cognitive impairments in Chinese patients with stroke and provided a theoretical basis for its clinical application in China. The Mandarin version of BoCA demonstrates good reliability and validity among community-dwelling stroke survivors with basic completion of testing functions and can be used as a supplementary tool for cognitive assessment.

## Data Availability

The raw data supporting the conclusions of this article will be made available by the authors, without undue reservation.
